# Prognostic Impact of Cytomegalovirus Reactivation After Transplantation From Cord Blood Compared to Other Donor Sources in Patients With Adult T‐Cell Leukemia/Lymphoma in the Pre‐Letermovir Era

**DOI:** 10.1111/tid.70070

**Published:** 2025-07-08

**Authors:** Takuya Fukushima, Hidehiro Itonaga, Hikaru Sakamoto, Wataru Takeda, Masahito Tokunaga, Takeharu Kato, Takuro Kuriyama, Toshiro Kawakita, Machiko Fujioka, Yasuhiko Miyazaki, Naoyuki Uchida, Yasuo Mori, Hirohisa Nakamae, Masao Ogata, Kazunori Imada, Makoto Onizuka, Kazuho Morichika, Yoshinobu Kanda, Takahiro Fukuda, Yoshiko Atsuta, Shigeo Fuji, Makoto Yoshimitsu, ATL Working Group of the Japanese Society for Transplantation and Cellular Therapy

**Affiliations:** ^1^ Laboratory of Hematoimmunology Department of Clinical Laboratory Sciences School of Health Sciences Faculty of Medicine University of the Ryukyus Okinawa Japan; ^2^ Transfusion and Cell Therapy Unit, Nagasaki University Hospital Nagasaki Japan; ^3^ Department of Hematology Nagasaki University Hospital Nagasaki Japan; ^4^ Department of Hematopoietic Stem Cell Transplantation National Cancer Center Hospital Tokyo Japan; ^5^ Department of Hematology Imamura General Hospital Kagoshima Japan; ^6^ Department of Hematology Hamanomachi Hospital Fukuoka Japan; ^7^ Department of Hematology NHO Kumamoto Medical Center Kumamoto Japan; ^8^ Department of Hematology Sasebo City General Hospital Sasebo Japan; ^9^ Department of Hematology Oita Prefectural Hospital Oita Japan; ^10^ Department of Hematology Federation of National Public Service Personnel Mutual Aid Associations Toranomon Hospital Tokyo Japan; ^11^ Hematology, Oncology & Cardiovascular Medicine, Kyushu University Hospital Fukuoka Japan; ^12^ Department of Hematology Osaka Metropolitan University Hospital Osaka Japan; ^13^ Department of Hematology Oita University Hospital Yufu Japan; ^14^ Department of Hematology Japanese Red Cross Osaka Hospital Osaka Japan; ^15^ Department of Hematology/Oncology Tokai University School of Medicine Isehara Japan; ^16^ Second Department of Internal Medicine Division of Endocrinology Diabetes and Metabolism, University of the Ryukyus Hospital Okinawa Japan; ^17^ Division of Hematology Jichi Medical University Shimotsuke Japan; ^18^ Japanese Data Center for Hematopoietic Cell Transplantation Nagakute Japan; ^19^ Department of Registry Science for Transplant and Cellular Therapy Aichi Medical University School of Medicine Nagakute Japan; ^20^ Department of Hematology Osaka International Cancer Center Institute Osaka Japan; ^21^ Department of Hematology and Rheumatology Kagoshima University Hospital Kagoshima Japan

**Keywords:** adult T‐cell leukemia/lymphoma, allogeneic hematopoietic stem cell transplantation, cytomegalovirus

## Abstract

**Background:**

Cytomegalovirus reactivation (CMV‐react) is an indicator for the worse non‐relapse mortality (NRM) and overall survival (OS) after allogeneic hematopoietic stem cell transplantation using HLA‐matched related donor (MRD) and unrelated donor (URD) for adult T‐cell leukemia/lymphoma (ATL). However, it remains unclear whether CMV‐react correlates with outcomes after unrelated cord blood (U‐CB) transplantation.

**Methods:**

We conducted a retrospective nationwide study to evaluate the impact of CMV‐react on the outcomes after posttransplant 100 days. Data were collected from 205, 461, and 268 patients who used MRD, URD, and U‐CB, respectively, between 2001 and 2022 and survived without relapse for over 100 days after transplantation.

**Results:**

In multivariate analyses, CMV‐react correlated with worse OS in the MRD (hazard ratio [HR], 1.56; 95% confidence interval [CI], 1.02–2.39; *p* = 0.04) and URD groups (HR, 1.45; 95% CI, 1.00–2.09; *p* = 0.05), but not in the U‐CB group (HR, 1.34; 95% CI, 0.88–2.03; *p* = 0.2). CMV‐react correlated with higher NRM in the MRD (HR, 1.79; 95% CI, 1.01–3.16; *p* = 0.05) and URD groups (HR, 1.68; 95% CI, 1.01–2.82; *p* = 0.05), but not in the U‐CB group (HR, 1.16; 95% CI, 0.62–2.19; *p* = 0.6). CMV‐react did not correlate with the incidence of relapse in any group.

**Conclusion:**

CMV‐react was not associated with the outcomes in the U‐CB group, while CMV‐react correlates with worse OS and NRM in the MRD and URD groups, indicating the need for a more intensive strategy for late‐phase complications in U‐CB transplantation for ATL with and without CMV‐react.

## Graphical abstract

Cytomegalovirus reactivation (CMV‐react) is an indicator to predict the increased non‐relapse mortality and decreased overall survival in adult T‐cell leukemia‐lymphoma patients who received allogeneic transplantation from HLA‐matched related and unrelated donors. But any prognostic impact of CMV‐react was not observed in patients after unrelated cord blood transplantation.

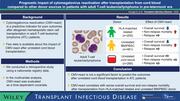

## 1 Introduction

Adult T‐cell leukemia/lymphoma (ATL) is a peripheral T‐lymphocytic neoplasm caused by human T‐cell leukemia virus Type I (HTLV‐1) [1, 2, 3, 4] that has different clinical manifestations from and a poorer prognosis than non‐Hodgkin's lymphomas [5, 6]. ATL is categorized into four clinical subtypes: the acute, lymphoma, chronic, and smoldering types [7, 8]. The former two subtypes are regarded as aggressive ATL, the median survival of which is 8–11 months [9, 10]. Multi‐agent chemotherapy and novel molecular target drugs are therapeutic options for aggressive ATL [9, 10, 11, 12, 13, 14, 15, 16], but do not provide durable remission. Allogeneic hematopoietic stem cell transplantation (allo‐HSCT) offers the only potential for a cure along with graft‐versus‐ATL effects [17, 18, 19, 20, 21, 22]. Transplantation from alternative donor sources, such as unrelated cord blood (U‐CB) and a human leukocyte antigen (HLA)‐haploidentical related donor, is now being increasingly performed to treat ATL [23, 24, 25, 26, 27, 28, 29, 30].

Cytomegalovirus reactivation (CMV‐react) is one of the most frequent infectious complications of allo‐HSCT, the incidence of which was reported to be approximately 80% among posttransplant patients [31, 32, 33, 34, 35]. Recent studies showed that CMV‐react correlated with marked reductions in the risk of posttransplant relapse in patients with acute myeloid leukemia (AML) and low‐risk myelodysplastic syndrome (MDS) [36, 37, 38, 39, 40], but not in those with acute lymphoblastic leukemia, high‐risk MDS, and malignant lymphoma [39, 40, 41, 42, 43]. Accordingly, the prognostic impact of CMV‐react on the risk of posttransplant relapse may differ with the type of underlying disease. We previously reported that CMV‐react was associated with an increase in non‐relapse mortality (NRM) after the posttransplant 100 days, leading to worse overall survival (OS) in ATL patients who underwent allo‐HSCT from an HLA‐matched related donor (MRD) and unrelated bone marrow/peripheral blood stem cell donor (URD) [34, 44].

Due to the lack of transfer of memory immune cells from donor to recipient and delayed immune recovery after U‐CB transplantation, the risk of viral infection is higher after transplantation with U‐CB than with MRD [45, 46, 47]. Regarding the prognostic impact of CMV‐react after U‐CB transplantation, CMV‐react was associated with a reduced risk of relapse in high‐risk acute leukemia, but increased NRM in standard‐risk acute leukemia [48, 49]. Therefore, CMV‐react after U‐CB transplantation has a distinct prognostic impact according to the disease type and risk. However, few studies have focused on the prognostic impact of CMV‐react by donor source, including U‐CB, in ATL patients.

To clarify the prognostic impact of CMV‐react on the outcomes after the posttransplant 100 days by donor source in ATL patients, we herein conducted a retrospective study on the largest cohort of ATL patients (*n* = 934) in the real‐world setting, using the Transplant Registry Unified Management Program (TRUMP) database.

### 1.1 Patients and Methods

#### 1.1.1 Data Collection

Data were collected by the Japanese Society for Transplantation and Cellular Therapy and the Japanese Data Center for Hematopoietic Cell Transplantation using TRUMP [50, 51, 52]. Data on these patients were collected and updated as of December 31, 2023. This study was approved by the TRUMP Committee (approval no. 13–20) and by the Ethics Committee of Nagasaki University Hospital (approval no. 24120502) at which this study was organized.

### 1.2 Inclusion Criteria

The original dataset consisted of 934 adult patients (aged 16 years or older) with ATL (i) who underwent first allo‐HSCT between January 1, 2001, and December 31, 2022, (ii) who used HLA‐MRD, URD, or single U‐CB, and (iii) who were CMV seropositive. We excluded patients who used HLA haploidentical donors and those who used prophylactic letermovir after allo‐HSCT. Data included clinical characteristics, such as age at transplantation, sex, disease subtype according to the Shimoyama classification [7], disease status at transplantation, date of transplantation, time from diagnosis to allo‐HSCT, HLA identity assessed by serological or molecular typing for HLA‐A, ‐B, and ‐DR loci, the source of stem cells [53], conditioning regimens, the incidence and severity of acute graft‐versus‐host disease (GVHD) and chronic GVHD, date last known alive, and the date and cause of death. Two physicians (Hidehiro Itonaga and Takuya Fukushima) independently reviewed the quality of collected data.

The present study did not include 506 patients from the original TRUMP dataset, because these patients experienced early death or disease relapse before Day 100 after allo‐HSCT (Table S1). Early death and disease relapse before Day 100 after allo‐HSCT are markedly affected by conditioning regimen‐related toxicities and the pretransplantation disease status; and persistent ATL disease and salvage therapy are likely to induce CMV‐react [54, 55]. In these terms, we limited the analysis to patients who survived without the relapse of ATL until Day 100 after allo‐HSCT as previously reported [39, 40, 43, 44].

### 1.3 Definitions

Japanese guidelines recommend the surveillance of pp65 antigenemia using an enzyme‐labeled antibody assay or standardized quantitative polymerase chain reaction (qPCR) to detect CMV. Since the qPCR method has been covered by the national health insurance system of Japan since August 2020, pp65 antigenemia has generally been performed as a clinical practice for allo‐HSCT at each institution in this study. CMV‐react was defined as the diagnosis of CMV antigenemia and/or CMV disease as previously described. Preemptive therapy was employed according to the protocol of each participating institute. Preemptive therapy was generally initiated when at least two CMV pp65 antigen‐positive cells per 50 000 white blood cells were identified [56].

Conditioning regimens were classified as myeloablative and reduced intensity (RIC) according to established criteria [57, 58]. GVHD prophylaxis was either a cyclosporine‐ or tacrolimus‐based regimen. The diagnosis and clinical grading of acute and chronic GVHD were performed according to standard criteria [59, 60].

In some patients, especially who undergo U‐CB transplantation, prophylactic foscavir is used to prevent human herpes virus‐6 encephalitis: for instance, foscavir daily 90 mg/kg intravenous infusion from Days 7 to 27 after allo‐HSCT [61]. Prophylactic foscavir was initiated in accordance with the institutional strategy.

### 1.4 Statistical Analysis

Continuous variables were compared using the Wilcoxon rank‐sum test or Kruskal–Wallis test. Categorical variables were compared between groups using the chi‐squared test. The probabilities of OS were estimated by the Kaplan–Meier method and group comparisons were performed by the Log‐rank test. The cumulative incidence of relapse (CIR) and NRM were estimated in a competing risk setting, and group comparisons were performed by the Gray test. Death before relapse was the competing event for CIR, while that for NRM was death after relapse [62, 63]. To investigate variables potentially affecting posttransplant outcomes, OS was evaluated using Cox proportional hazards regression models, and CIR and NRM were examined using the Fine and Gray proportional hazards model for the subdistribution of competing risks [63]. In these models, CMV‐react and acute/chronic GVHD were evaluated as time‐dependent covariates [64].

Factors associated with at least borderline significance (*p* ≤ 0.10) in the univariate analysis, CMV‐react, and acute GVHD were subjected to a multivariate analysis using a backward stepwise covariate selection. Effect estimates were expressed as hazard ratios (HRs) with 95% confidence intervals (95% CIs). All *p* values were two‐tailed, and *p* values ≤ 0.05 were considered to be significant. Since the test for interactions may be underpowered, a threshold of ≤ 0.20 for *p*_interaction_ was used to indicate significant differences across prespecified subgroups. All statistical analyses were performed using Stata software, version 12 (Stata, College Station, TX, USA), and graphical presentations were performed using EZR software, version 1.24 (Saitama Medical Center, Jichi Medical University) [65].

## 2 Results

### 2.1 Patient Characteristics and Transplant Procedures

Patient characteristics are shown in Table 1. Donor sources for transplantation were as follows: MRD in 205 patients (the MRD group), URD in 461 (the URD group), and U‐CB in 268 (the U‐CB group). Median patient ages at allo‐HSCT in the MRD, URD, and U‐CB groups were 54 years (range, 23–70 years), 56 years (range, 26–72 years), and 58 years (range, 24–78 years), respectively. Regarding CMV seropositivity in donors, 152 (74.1%) and 248 (53.8%) were detected in the MRD and URD groups, respectively (*p* < 0.01). The U‐CB group was more likely to receive the RIC regimen than the other groups (*p* = 0.04). The prophylactic use of foscavir is more frequently observed in the U‐CB group (*n* = 36, 13.4%) than in the MRD (*n* = 2, 1.0%) and URD groups (*n* = 6, 1.3%) (*p* < 0.01). The cumulative incidence of acute GVHD at 100 days in the MRD, URD, and U‐CB groups were 69.3% (62.5%–75.1%), 73.8% (69.5%–77.5%), and 66.3% (60.3%–71.6%) (*p* = 0.2), respectively. The cumulative incidence of chronic GVHD at 3 years were 51.8% (44.7%–58.4%), 40.2% (35.6%–44.7%), and 25.2% (20.2%–30.6%) (*p* < 0.01), respectively.

**TABLE 1:** Patient characteristics.

	MRD		URD		U‐CB		
Total	205		461		268		*p*
Median age at allo‐HSCT (range), years	54 (23–70)		56 (26–72)		58 (24–78)		<0.001
< 50	7 0	(34.1%)	92	(20.0%)	45	(16.8%)	<0.001
≥ 50	135	(65.9%)	369	(80.0%)	223	(83.2%)	
Patient sex							
Male	106	(51.7%)	235	(51.0%)	142	(53.0%)	0.872
Female	99	(48.3%)	226	(49.0%)	126	(47.0%)	
PS at allo‐HSCT							
0–1	190	(92.7%)	437	(95.2%)	247	(95.4%)	0.346
2–4	15	(7.3%)	22	(4.8%)	12	(4.6%)	
HCT‐CI							
0	102	(49.8%)	279	(60.5%)	170	(63.4%)	<0.001
1–2	39	(19.0%)	76	(16.5%)	50	(18.7%)	
≥ 3	15	(7.3%)	56	(12.1%)	22	(8.2%)	
Missing	49	(23.9%)	50	(10.8%)	26	(9.7%)	
Disease status at HSCT							
CR	95	(46.3%)	236	(51.2%)	116	(43.3%)	0.149
Others	94	(45.9%)	183	(39.7%)	119	(44.4%)	
Missing	16	(7.8%)	42	(9.1%)	33	(12.3%)	
Clinical subtype							
Acute type	125	(61.0%)	275	(59.7%)	174	(64.9%)	0.017
Others	79	(38.5%)	183	(39.7%)	86	(32.1%)	
Missing	1	(0.5%)	3	(0.7%)	8	(3.0%)	
Sex combination							
Others	158	(77.1%)	397	(86.3%)	184	(72.4%)	<0.001
Female donor to male recipient	47	(22.9%)	63	(13.7%)	70	(27.6%)	
Donor's CMV serostatus							
Negative	20	(9.8%)	168	(36.4%)	—		<0.001
Positive	152	(74.1%)	248	(53.8%)	—		
Missing	33	(16.1%)	45	(9.8%)			
Donor's HTLV‐1 serostatus							
Negative	109	(53.2%)	461	(100.0%)	268	(100.0%)	<0.001
Positive	57	(27.8%)	0	(0.0%)	0	(0.0%)	
Missing	39	(19.0%)	0	(0.0%)	0	(0.0%)	
Conditioning regimen intensity							
MAC	92	(44.9%)	178	(38.6%)	90	(33.6%)	0.044
RIC	113	(55.1%)	283	(61.4%)	178	(66.4%)	
GVHD prophylaxis							
CsA‐based	175	(85.4%)	55	(12.0%)	81	(30.6%)	<0.001
Tac‐based	30	(14.6%)	399	(87.3%)	182	(68.7%)	
Others	0	(0.0%)	3	(0.7%)	2	(0.8%)	
Interval between diagnosis and allo‐HSCT							
< 120 days	56	(27.3%)	16	(3.5%)	91	(34.0%)	<0.001
≥ 120 days	149	(72.7%)	445	(96.5%)	177	(66.0%)	
Years of allo‐HSCT							
2001–2011	126	(61.5%)	194	(42.1%)	91	(34.0%)	<0.001
2012–2022	79	(38.5%)	267	(57.9%)	177	(66.0%)	

Abbreviations: allo‐HSCT, allogeneic hematopoietic stem cell; CMV, cytomegalovirus; CR, complete remission; GVHD, graft‐versus‐host disease; HCT‐CI, hematopoietic cell transplantation‐specific comorbidity index; HTLV‐1, human T‐cell lymphotropic virus type I; MAC, myeloablative conditioning; MRD, human leukocyte antigen‐matched related donor; PS, performance status; RIC, reduced intensity conditioning; U‐CB, unrelated cord blood; URD, unrelated bone marrow/peripheral blood stem cell donor.

### 2.2 CMV Reactivation After Allo‐HSCT

**FIGURE 1:**
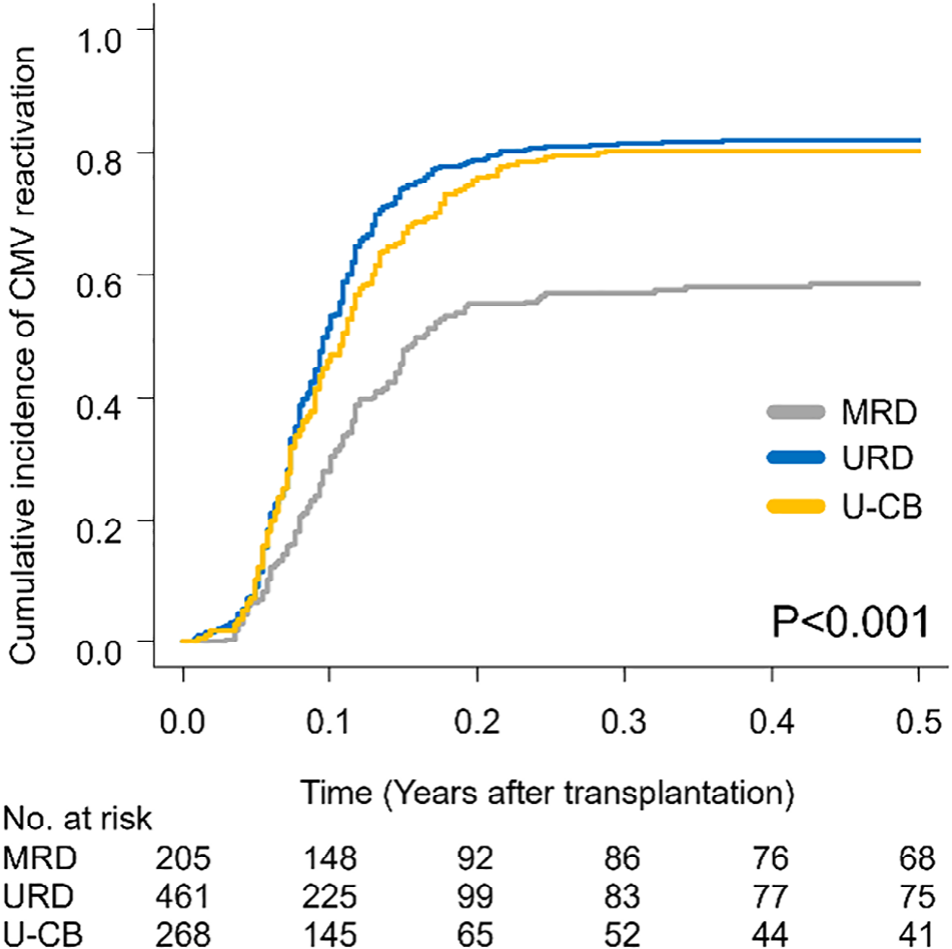
CMV reactivation after transplantation by donor source. CMV, cytomegalovirus; MRD, human leukocyte antigen‐matched related donor; URD, unrelated bone marrow/peripheral blood stem cell donor; U‐CB, unrelated cord blood.

The cumulative incidence of CMV‐react on Day 100 after allo‐HSCT in the MRD, URD, and U‐CB groups were 57.0% (95% CI, 50.0%–63.5%), 82.2% (95% CI, 78.4%–85.4%), and 80.2% (95% CI, 74.9%–84.5%), respectively (*p* < 0.01) (Figure 1). In the entire population (*n* = 934), CMV antigenemia was only observed in 717 patients (76.8%), and CMV diseases, such as gastroenteritis, hepatitis, pneumonia, retinitis, and encephalitis, were detected in 105 patients (11.2%) (Table S2). No significant differences were observed in the profile of CMV‐react by donor source. In all three groups, the development of acute GVHD was associated with CMV‐react (*p* < 0.01).

### 2.3 Impact of CMV Reactivation on OS

**FIGURE 2:**
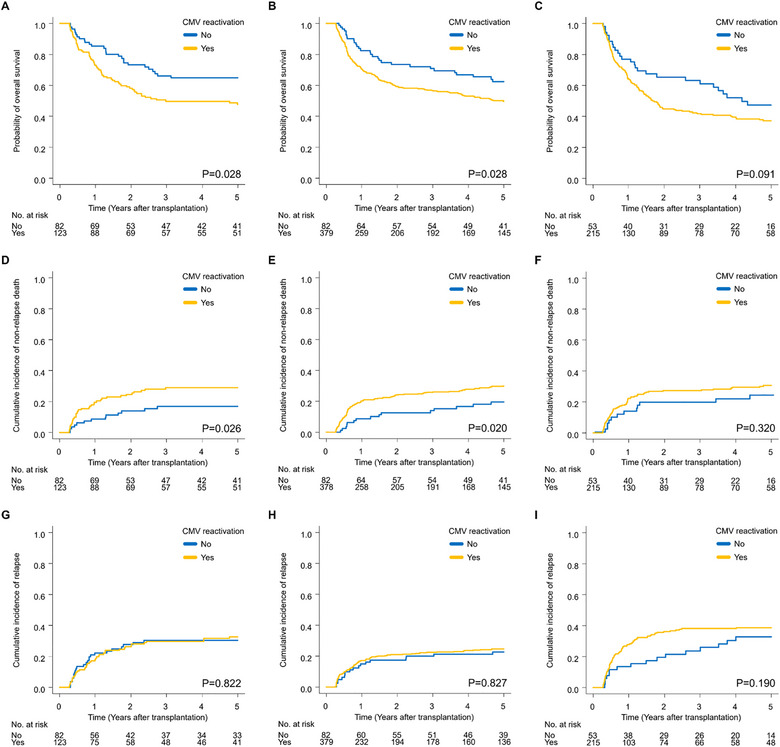
Posttransplant outcomes by donor source. Overall survival (OS) in the MRD (A), URD (B), and U‐CB groups (C). Non‐relapse mortality (NRM) in the MRD (D), URD (E), and U‐CB (F) groups. Cumulative incidence of relapse (CIR) in the MRD (G), URD (H), and U‐CB groups (I).

Among patients who survived without relapse for more than 100 days after allo‐HSCT, univariate analyses showed worse OS among patients with CMV‐react than those without CMV‐react in the MRD (49.6% vs. 66.1% at 3 years, *p* = 0.03) and URD groups (56.6% vs. 70.8% at 3 years, *p* = 0.03) (Figure 2A,B). In the U‐CB group, OS was similar in patients with and without CMV‐react (41.7% vs. 63.1% at 3 years, *p* = 0.091) (Figure 2C). Multivariate analyses revealed that OS was significantly worse in patients with than in those without an episode of CMV‐react in the MRD (HR, 1.56; 95% CI, 1.02–2.39; *p* = 0.04) and URD groups (HR, 1.45; 95% CI, 1.00–2.09; *p* = 0.05), but not in the U‐CB group (HR, 1.34; 95% CI, 0.88–2.03; *p* = 0.2) (Table 2 and Tables S3–S5). To identify the acute GVHD‐associated subgroup in which CMV‐react resulted in further disadvantages, we examined the interaction effect between CMV‐react and acute GVHD. No significant interaction effect was noted between CMV‐react and acute GVHD in the MRD (*p*_interaction_ = 0.49), URD (*p*_interaction_ = 0.37), and U‐CB group (*p*_interaction_ = 0.73).

**TABLE 2:** Multivariate analysis of the prognostic impact of CMV reactivation.

Donor	Outcome	CMV reactivation	HR	(95% CI)	*p* value
MRD	Overall mortality	No	1.00		
		Yes	1.56	(1.02–2.39)	0.041
	Relapse	No	1.00		
		Yes	0.99	(0.59–1.68)	0.983
	NRM	No	1.00		
		Yes	1.79	(1.01–3.16)	0.047
URD	Overall mortality	No	1.00		
		Yes	1.45	(1.00–2.09)	0.048
	Relapse	No	1.00		
		Yes	1.16	(0.71–1.91)	0.552
	NRM	No	1.00		
		Yes	1.68	(1.01–2.82)	0.048
U‐CB	Overall mortality	No	1.00		
		Yes	1.34	(0.88–2.03)	0.177
	Relapse	No	1.00		
		Yes	1.56	(0.92–2.66)	0.100
	NRM	No	1.00		
		Yes	1.16	(0.62–2.19)	0.646

Abbreviations: CI, confidence interval; HR, hazard ratio; NRM, non‐relapse morality.

### 2.4 Impact of CMV Reactivation on NRM

We investigated the impact of CMV‐react on NRM because previous studies reported a relationship between CMV‐react and increased NRM [34, 44]. Univariate analyses revealed a higher rate of NRM in patients with CMV‐react than in those without CMV‐react in the MRD (28.8% vs. 16.8% at 3 years, *p* = 0.03) and URD groups (25.8% vs. 13.9% at 3 years, *p* = 0.02), whereas it was similar in the U‐CB group (27.2% vs. 19.2% at 3 years, *p* = 0.3) (Figure 2D–F). Multivariate analyses showed a significant increase in NRM in the MRD (HR, 1.79; 95% CI, 1.01–3.16; *p* = 0.05) and URD groups (HR, 1.68; 95% CI, 1.01–2.82; *p* = 0.05), but not in the U‐CB group (HR, 1.16; 95% CI, 0.62–2.19; *p* = 0.6) (Table 2 and Tables S3–S5). No significant interaction effect was detected between CMV‐react and acute GVHD in the MRD (*p*_interaction_ = 0.44), URD (*p*_interaction_ = 0.27), and U‐CB groups (*p*_interaction_ = 0.64).

### 2.5 Impact of CMV Reactivation on Relapse

Univariate analyses revealed similar CIR between patients with and without CMV‐react in the MRD (29.7% vs. 30.4% at 3 years, *p* = 0.8), URD (22.4% vs. 20.0% at 3 years, *p* = 0.8), and U‐CB groups (38.0% vs. 23.7% at 3 years, *p* = 0.2) (Figure 2G–I). Multivariate analyses showed no significant difference between patients with and without CMV‐react in all three groups (Table 2 and Tables S3–S5).

### 2.6 Causes of Death by Donor Source

The causes of death are shown in Table 3. In all three groups, the recurrence of ATL was the most frequent cause of death. Infection was the most frequent cause of non‐relapse death: 15 out of 107 patients (14.0%) in the MRD group, 47 out of 238 (19.7%) in the URD group, and 27 out of 167 (16.2%) in the U‐CB group. Regarding the pathogen causing infection‐related death, the most frequent was bacteria and/or fungi in the three groups: 10 (9.3%), 30 (12.6%), and 14 patients (8.4%) in the MRD, URD, and U‐CB groups, respectively. Viral infection‐related death occurred in two (1.9%), six (2.5%), and eight patients (4.8%) in the MRD, URD, and U‐CB groups, respectively. CMV infection‐related death was detected in zero (0.0%), three (1.3%), and three patients (1.8%) in the MRD, URD, and U‐CB groups, respectively.

**TABLE 3:** Cause of death.

	MRD			URD			U‐CB	
Total	107			238			167	
Relapse or progression of ATL	46	(43.0%)		100	(42.0%)		86	(51.5%)
Infection	15	(14.0%)		47	(19.7%)		27	(16.2%)
Organ failure	9	(8.4%)		29	(12.2%)		13	(7.8%)
GVHD	14	(13.1%)		23	(9.7%)		9	(5.4%)
Idiopathic pulmonary syndrome	10	(9.3%)		16	(6.7%)		11	(6.6%)
Bleeding	2	(1.9%)		4	(1.7%)		1	(0.6%)
SOS	1	(0.9%)		0	(0.0%)		1	(0.6%)
Secondary malignancy	3	(2.8%)		6	(2.5%)		9	(5.4%)
TMA	2	(1.9%)		6	(2.5%)		2	(1.2%)
Other	5	(4.7%)		7	(2.9%)		8	(4.8%)

Abbreviations: ATL, adult T‐cell leukemia/lymphoma; SOS, sinusoidal obstruction syndrome; TMA, thrombotic microangiopathy.

### 2.7 Impact of CMV Reactivation in U‐CB Transplantation With Prophylactic Foscavir

Since foscavir is used to prevent human herpes virus‐6 encephalitis in some patients who undergo U‐CB transplantation [61], we examined the prognostic impact of CMV‐react in patients using prophylactic foscavir (*n* = 36). In univariate analyses of patients who used prophylactic foscavir after U‐CB transplantation, no significant differences between those with and without CMV‐react were observed in OS (47.6% vs. 70.8% at 3 years, *p* = 0.8), NRM (29.0% vs. 17.4% at 3 years, *p* = 0.9), or CIR (34.7% vs. 23.3% at 3 years, *p* = 0.8).

## 3 Discussion

The present study investigated the prognostic impact of CMV‐react in ATL patients. Since NRM was previously shown to be higher in patients with ATL than in those with other hematological neoplasms [34], it is important to note predictive indicators for NRM in the posttransplant setting. Moreover, the NRM rate was more likely to be higher in the U‐CB group than in the MRD and URD groups with ATL. This retrospective study using a large cohort enabled us to estimate more accurately the impact of CMV‐react on the outcomes (OS, CIR, and NRM) after the posttransplant 100 days by donor sources, and provided detailed information on its prognostic impact on U‐CB transplantation for ATL.

The most significant result is that CMV‐react did not correlate with posttransplant outcomes in the U‐CB group, but correlated with worse NRM and OS in the MRD and URD groups. Bacterial/fungal infection‐related death was the most frequent cause of death in all three groups, while viral infection‐related death was more likely to be observed in the U‐CB group (4.8%) than in the MRD (1.9%) and URD groups (2.5%). Although the significance of these differences was not clear due to small number of patients who died of viral infection‐related death, one possible reason for the similar outcomes of patients with and without CMV‐react in the U‐CB group may be high susceptibility to viral infections, as previously reported [19, 26, 44, 66]. In addition, although the frequency of GVHD‐associated death was low, deaths due to organ failure and idiopathic pulmonary disease were still issues in the U‐CB group. Another possible explanation is that the high incidence of early death and disease relapse may have attenuated the prognostic significance of CMV‐react in the U‐CB group. Based on these results, comprehensive careful supportive care after the posttransplant 100 days is necessary for ATL patients regardless of CMV‐react in the practical setting of U‐CB transplantation.

In the setting of U‐CB transplantation, the present results on ATL differed from previous findings on acute leukemia and MDS. CMV‐react had a negative impact on NRM in patients with acute leukemia and MDS [48], but not in those with ATL. Previous studies suggested that persistent compromised cellular immunity after transplantation increased susceptibility to CMV and other infections, leading to a high rate of NRM. The lack of a significant difference in the frequency of infection‐associated death between patients with and without CMV‐react in the U‐CB group suggests that U‐CB transplantation harbors a persistent cellular immune deficiency that is not always associated with CMV‐react in ATL patients. These hypotheses need to be examined in further studies that assess immune reconstitution.

Another significant result was that CMV‐react did not affect the incidence of relapse after the posttransplant 100 days in the U‐CB group. In high‐risk AML and MDS after U‐CB transplantation, an increase in HLA‐Cw expression after CMV‐react was suggested to increase the number of natural killer (NK) cells, thereby reducing the risk of relapse [49, 67]. HLA‐Cw1/Cw2 (killer cell immunoglobulin‐like receptor 2DL‐ligand) mismatch has been reported to further enhance immunological effects against AML and MDS along with CMV‐react. Therefore, future research on the impact of NK cell‐mediated graft‐versus ATL effects with CMV‐react in U‐CB transplantation for ATL is needed because innate immune effector cells, including NK cells, may exhibit potent cytotoxic activities against HTLV‐1‐infected cells [68].

In the MRD and URD groups, CMV‐react was associated with lower OS and higher NRM after the posttransplant 100 days, which is consistent with the results of the previous studies [34, 44]. These results indicate the significance of optimal management for CMV‐react in the MRD and URD groups. Recent advances in the therapeutic options for the posttransplant CMV‐react will contribute to the improved outcomes. For instance, extended duration of letermovir prophylaxis should be considered in patients who have GVHD requiring systemic cortico‐steroid administration [69]. Moreover, early indication of maribavir would improve the clinical course of refractory or resistant CMV‐react with minimizing hematopoietic and organ toxicities [70, 71]. Future study is necessary to examine whether these novel modalities truly improve the posttransplant outcomes among ATL patients who develop CMV‐react.

The present study has some limitations, which were mainly due to its retrospective nature using registry data and the small number of ATL patients. Although we carefully evaluated the eligibility of patients, baseline characteristics and transplant procedures were highly heterogenous, which may have introduced a selection bias. Furthermore, detailed information was not available on anti‐bacterial and ‐fungal prophylaxis and intentional medication to induce graft‐versus‐ATL effects (e.g., the early withdrawal of GVHD prophylaxis); therefore, the present results need to be interpreted with caution and confirmed in larger studies including more factors. Furthermore, we were unable to assess several potential prognostic factors due to the lack of clinical data (e.g., serum albumin levels, calcium levels, soluble interleukin‐2 receptor levels, and gene mutational profiles) [72, 73, 74, 75] and detailed information on the disease status (complete response, partial remission, stable disease, and progressive disease) at allo‐HSCT in the TRUMP database.

In conclusion, the present study demonstrated that CMV‐react had no prognostic impact on the posttransplant outcomes after 100 days following U‐CB transplantation for ATL, although CMV‐react correlated with worse OS and NRM after the posttransplant 100 days in the setting of allo‐HSCT from MRD and URD. Since allo‐HSCT offers the best opportunity for a curative outcome along with graft‐versus‐ATL effects, it is important to develop optimized supportive care for ATL patients that will minimize NRM. The present results suggest that intensified management for infection‐ and GVHD‐associated complications is required in U‐CB transplantation for ATL with and without CMV‐react. This strategy may reduce NRM in U‐CB transplantation for ATL.

## Author Contributions

**Takuya Fukushima**: conceptualization, writing – original draft, formal analysis, data curation. **Hidehiro Itonaga**: conceptualization, writing – original draft, investigation, data curation, formal analysis. **Hikaru Sakamoto**: writing – review and editing. **Wataru Takeda**: writing – review and editing. **Masahito Tokunaga**: writing – review and editing. **Takeharu Kato**: writing – review and editing. **Takuro Kuriyama**: writing – review and editing. **Toshiro Kawakita**: writing – review and editing. **Machiko Fujioka**: writing – review and editing. **Yasuhiko Miyazaki**: writing – review and editing. **Naoyuki Uchida**: writing – review and editing. **Yasuo Mori**: writing – review and editing. **Hirohisa Nakamae**: writing – review and editing. **Masao Ogata**: writing – review and editing. **Kazunori Imada**: writing – review and editing. **Makoto Onizuka**: writing – review and editing. **Kazuho Morichika**: writing – review and editing. **Yoshinobu Kanda**: writing – review and editing, supervision. **Takahiro Fukuda**: writing – review and editing. **Yoshiko Atsuta**: writing – review and editing, resources, supervision. **Shigeo Fuji**: writing – review and editing, supervision. **Makoto Yoshimitsu**: supervision, writing – review and editing.

## Supporting information




**Supplementary Table 1**: Survival status of patients who died or developed relapse within day 100.
**Supplementary Table 2**: CMV reactivation by donor source.
**Supplementary Table 3**: Multivariate analysis of the MRD group.
**Supplementary Table 4**: Multivariate analysis of the URD group.
**Supplementary Table 5**: Multivariate analysis of the U‐CB group.

## Data Availability

All data are available within the article or supplementary files or upon reasonable request from the corresponding author Hidehiro Itonaga (itoman820hide@outlook.jp).
