# Supplementary material for: Prognostic Impact of Cytomegalovirus Reactivation After Transplantation From Cord Blood Compared to Other Donor Sources in Patients With Adult T‐Cell Leukemia/Lymphoma in the Pre‐Letermovir Era
Source: Transpl Infect Dis. 2025 Jul 8;27(5):e70070. doi: 10.1111/tid.70070 (PMC12519938; doi:10.1111/tid.70070)
Supplement: Supplementary file 1 — Supplementary Table 1: Survival status of patients who died or developed relapse within day 100. Supplementary Table 2: CMV reactivation by donor source. Supplementary Table 3: Multivariate analysis of the MRD group. Supplementary Table 4: Multivariate analysis of the URD group. Supplementary Table 5: Multivariate analysis of the U‐CB group. [file TID-27-e70070-s001.docx]

**Supplementary Table 1. Survival status of patients who died or developed relapse within day 100**

| Donor | Survival status at day100 | CMV reactivation (-) | | CMV reactivation (+) | | P-value |
| --- | --- | --- | --- | --- | --- | --- |
| MRD | Total | n=45 | | n=50 | | 0.92 |
|  | Alive after relapse | 6 | (13.3%) | 5 | (10.0%) |  |
|  | Death after relapse | 23 | (51.1%) | 27 | (54.0%) |  |
|  | Death without relapse | 16 | (35.6%) | 18 | (36.0%) |  |
| URD | Total | n=122 | | n=96 | | 0.22 |
|  | Alive after relapse | 16 | (13.1%) | 7 | (7.3%) |  |
|  | Death after relapse | 36 | (29.5%) | 37 | (38.5%) |  |
|  | Death without relapse | 70 | (57.4%) | 52 | (54.2%) |  |
| U-CB | Total | n=109 | | n=80 | | 0.46 |
|  | Alive after relapse | 7 | (6.4%) | 6 | (7.5%) |  |
|  | Death after relapse | 35 | (32.1%) | 32 | (40.0%) |  |
|  | Death without relapse | 67 | (61.5%) | 42 | (52.5%) |  |

The original dataset included 300, 679, and 457 patients in the MRD, URD, and U-CB groups, respectively. According to the inclusion criteria, 95 out of 300 (31.7%), 218 out of 679 (32.1%), 189 out of 457 patients (41.4%) in the MRD, URD, and U-CB groups, respectively, were excluded from this study due to the early death and/or disease relapse before day 100. As for the cause of death without relapse among patients without CMV reactivation, bacterial infection was most frequently observed in the MRD group (n=4, 25.0%), the URD group (n=18, 25.7%), and the U-CB group (n=21, 31.3%). As for the cause of death without relapse among patients with CMV reactivation, acute graft-versus-host disease was most frequently observed in the MRD group (n=6, 33.3%); and idiopathic pulmonary syndrome was observed in the URD group (n=13, 25.0%) and the U-CB group (n=10, 23.8%). Three survival status were compared between patients with and without CMV reactivation, using the chi-squared test.

Abbreviations: human leukocyte antigen-matched related donor, MRD; unrelated bone marrow/peripheral blood stem cell donor, URD; unrelated cord blood, U-CB; cytomegalovirus, CMV.

**Supplementary Table 2. CMV reactivation by donor source**

|  | MRD | |  | URD | |  | U-CB | |
| --- | --- | --- | --- | --- | --- | --- | --- | --- |
| Total | 125 |  |  | 381 |  |  | 216 |  |
| Antigenemia | 108 | (86.4%) |  | 325 | (85.3%) |  | 184 | (85.2%) |
| Colitis | 8 | (6.4%) |  | 36 | (9.4%) |  | 12 | (5.6%) |
| Pneumoniae | 3 | (2.4%) |  | 2 | (0.5%) |  | 5 | (2.3%) |
| Hepatitis | 0 | (0.0%) |  | 3 | (0.8%) |  | 1 | (0.5%) |
| Retinitis | 4 | (3.2%) |  | 10 | (2.6%) |  | 8 | (3.7%) |
| Other | 2 | (1.6%) |  | 5 | (1.3%) |  | 6 | (2.8%) |

**Supplementary Table 3. Multivariate analysis of the MRD group**

|  | **Overall mortality** | | | | |  | **Relapse rate** | | | | |  | **Non-relapse mortality** | | | | |
| --- | --- | --- | --- | --- | --- | --- | --- | --- | --- | --- | --- | --- | --- | --- | --- | --- | --- |
|  | **Univariate analysis** | |  | **Multivariate analysis** | |  | **Univariate analysis** | |  | **Multivariate analysis** | |  | **Univariate analysis** | |  | **Multivariate analysis** | |
| **Variable** | **HR  (95% CI)** | ***P*** |  | **HR  (95% CI)** | ***P*** |  | **HR  (95% CI)** | ***P*** |  | **HR  (95% CI)** | ***P*** |  | **HR  (95% CI)** | ***P*** |  | **HR  (95% CI)** | ***P*** |
| **Age at allo-HSCT** |  |  |  |  |  |  |  |  |  |  |  |  |  |  |  |  |  |
| <50 years | 1.00 |  |  | 1.00 |  |  | 1.00 |  |  | - | |  | 1.00 |  |  | 1.00 |  |
| ≥50 years | 2.13 (1.36-3.35) | 0.001 |  | 1.92 (1.22-3.03) | 0.005 |  | 1.32 (0.79-2.20) | 0.296 |  | - | |  | 1.98 (1.10-3.55) | 0.023 |  | 1.84 (1.03-3.28) | 0.038 |
| **Patient sex** |  |  |  |  |  |  |  |  |  |  |  |  |  |  |  |  |  |
| Female | 1.00 |  |  | - | |  | 1.00 |  |  | - | |  | 1.00 |  |  | - | |
| Male | 1.22 (0.83-1.79) | 0.307 |  | *-* | |  | 0.99 (0.61-1.59) | 0.954 |  | - | |  | 1.50 (0.91-2.48) | 0.112 |  | - | |
| **PS at allo-HSCT** |  |  |  |  |  |  |  |  |  |  |  |  |  |  |  |  |  |
| 0-1 | 1.00 |  |  | *Not selected* | |  | 1.00 |  |  | - | |  | 1.00 |  |  | - | |
| 2-4 | 1.92 (1.03-3.59) | 0.041 |  |  |  |  | 0.71 (0.27-1.84) | 0.477 |  | - | |  | 1.95 (0.79-4.78) | 0.146 |  | - | |
| **HCT-CI** |  |  |  |  |  |  |  |  |  |  |  |  |  |  |  |  |  |
| 0 | 1.00 |  |  | 1.00 |  |  | 1.00 |  |  | - | |  | 1.00 |  |  | 1.00 |  |
| 1-2 | 2.09 (1.27-3.44) | 0.004 |  | 2.22 (1.33-3.72) | 0.002 |  | 0.58 (0.29-1.16) | 0.126 |  | - | |  | 3.97 (2.00-7.87) | <0.001 |  | 4.40 (2.22-8.72) | <0.001 |
| ≥3 | 2.39 (1.20-4.78) | 0.014 |  | 3.09 (1.49-6.42) | 0.002 |  | 0.66 (0.24-1.86) | 0.436 |  | - | |  | 3.81 (1.62-8.99) | 0.002 |  | 5.77 (2.26-14.74) | <0.001 |
| Missing | 1.52 (0.95-2.43) | 0.084 |  | 1.59 (0.98-2.59) | 0.060 |  | 0.61 (0.33-1.15) | 0.128 |  | - | |  | 2.46 (1.29-4.68) | 0.006 |  | 2.70 (1.38-5.29) | 0.004 |
| **Disease status** |  |  |  |  |  |  |  |  |  |  |  |  |  |  |  |  |  |
| CR | 1.00 |  |  | - | |  | 1.00 |  |  | - | |  | 1.00 |  |  | - | |
| Others | 1.11 (0.75-1.66) | 0.596 |  | - | |  | 1.14 (0.68-1.88) | 0.623 |  | - | |  | 1.27 (0.76-2.13) | 0.368 |  | - | |
| **Clinical subtype** |  |  |  |  |  |  |  |  |  |  |  |  |  |  |  |  |  |
| Acute | 1.00 |  |  | - | |  | 1.00 |  |  | - | |  | 1.00 |  |  | - | |
| Others | 0.92 (0.62-1.37) | 0.694 |  | - | |  | 0.79 (0.48-1.30) | 0.356 |  | - | |  | 1.09 (0.65-1.83) | 0.745 |  | - | |
| **Sex combination** |  |  |  |  |  |  |  |  |  |  |  |  |  |  |  |  |  |
| Others | 1.00 |  |  | 1.00 |  |  | 1.00 |  |  | - | |  | 1.00 |  |  | 1.00 |  |
| Female donor to male recipient | 1.73 (1.13-2.65) | 0.012 |  | 2.30 (1.46-3.63) | <0.001 |  | 0.73 (0.39-1.38) | 0.336 |  | - | |  | 2.45 (1.45-4.15) | 0.001 |  | 3.73 (2.16-6.45) | <0.001 |
| **Donor’s CMV serostatus** |  |  |  |  |  |  |  |  |  |  |  |  |  |  |  |  |  |
| Positive | 1.00 |  |  | - | |  | 1.00 |  |  | - | |  | 1.00 |  |  | - | |
| Negative | 1.35 (0.75-2.44) | 0.314 |  | - | |  | 1.73 (0.84-3.55) | 0.138 |  | - | |  | 1.16 (0.55-2.44) | 0.701 |  | - | |
| **Donor’s HTLV-1 serostatus** |  |  |  |  |  |  |  |  |  |  |  |  |  |  |  |  |  |
| Negative | 1.00 |  |  | - | |  | 1.00 |  |  | - | |  | 1.00 |  |  | - | |
| Positive | 1.05 (0.67-1.64) | 0.827 |  | - | |  | 1.04 (0.60-1.81) | 0.886 |  | - | |  | 1.01 (0.57-1.78) | 0.973 |  | - | |
| **Conditioning regimen intensity** |  |  |  |  |  |  |  |  |  |  |  |  |  |  |  |  |  |
| RIC | 1.00 |  |  | - | |  | 1.00 |  |  | - | |  | 1.00 |  |  | - | |
| MAC | 0.94 (0.64-1.38) | 0.749 |  | - | |  | 0.91 (0.57-1.48) | 0.713 |  | - | |  | 1.08 (0.65-1.78) | 0.769 |  | - | |
| **GVHD prophylaxis** |  |  |  |  |  |  |  |  |  |  |  |  |  |  |  |  |  |
| CsA-based | 1.00 |  |  | - | |  | 1.00 |  |  | - | |  | 1.00 |  |  | - | |
| Tac-based | 1.34 (0.79-2.29) | 0.280 |  | - | |  | 1.47 (0.79-2.74) | 0.225 |  | - | |  | 1.02 (0.47-2.22) | 0.958 |  | - | |
| **Interval from diagnosis to allo-HSCT** |  |  |  |  |  |  |  |  |  |  |  |  |  |  |  |  |  |
| <120 days | 1.00 |  |  | - | |  | 1.00 |  |  | 1.00 |  |  | 1.00 |  |  | *Not selected* | |
| ≥120 days | 1.12 (0.73-1.72) | 0.608 |  | - | |  | 0.64 (0.39-1.05) | 0.079 |  | 0.66 (0.39-1.12) | 0.125 |  | 1.69 (0.91-3.16) | 0.098 |  |  |  |
| **Years of allo-HSCT** |  |  |  |  |  |  |  |  |  |  |  |  |  |  |  |  |  |
| 2001-2011 | 1.00 |  |  | - | |  | 1.00 |  |  | 1.00 |  |  | 1.00 |  |  | - | |
| 2012-2022 | 1.11 (0.74-1.66) | 0.620 |  | - | |  | 1.83 (1.14-2.95) | 0.013 |  | 1,86 (1.15-3.02) | 0.012 |  | 0.84 (0.49-1.45) | 0.532 |  | - | |
| **Acute GVHD** |  |  |  |  |  |  |  |  |  |  |  |  |  |  |  |  |  |
| No | 1.00 |  |  | 1.00 |  |  | 1.00 |  |  | 1.00 |  |  | 1.00 |  |  | 1.00 |  |
| Grade I | 1.07 (0.61-1.90) | 0.804 |  | 0.82 (0.45-1.49) | 0.507 |  | 1.32 (0.68-2.54) | 0.411 |  | 1.36 (0.70-2.64) | 0.357 |  | 0.75 (0.34-1.63) | 0.463 |  | 0.47 (0.20-1.12) | 0.089 |
| Grade II-IV | 1.38 (0.88-2.15) | 0.160 |  | 1.16 (0.73-1.83) | 0.534 |  | 0.88 (0.51-1.52) | 0.648 |  | 0.97 (0.54-1.76) | 0.928 |  | 1.32 (0.74-2.37) | 0.350 |  | 1.04 (0.60-1.83) | 0.881 |
| **Chronic GVHD** |  |  |  |  |  |  |  |  |  |  |  |  |  |  |  |  |  |
| No | 1.00 |  |  | 1.00 |  |  | 1.00 |  |  | - | |  | 1.00 |  |  | 1.00 |  |
| Limited type | 0.55 (0.31-0.99) | 0.045 |  | 0.50 (0.28-0.89) | 0.020 |  | 1.10 (0.59-2.04) | 0.770 |  | - | |  | 1.10 (0.50-2.45) | 0.809 |  | 0.62 (0.27-1.44) | 0.266 |
| Extensive type | 0.83 (0.54-1.25) | 0.365 |  | 0.72 (0.47-1.11) | 0.133 |  | 0.98 (0.56-1.73) | 0.952 |  | - | |  | 2.25 (1.34-3.76) | 0.002 |  | 1.18 (0.70-2.00) | 0.526 |
| **CMV reactivation** |  |  |  |  |  |  |  |  |  |  |  |  |  |  |  |  |  |
| No | 1.00 |  |  | 1.00 |  |  | 1.00 |  |  | 1.00 |  |  | 1.00 |  |  | 1.00 |  |
| Yes | 1.54 (1.03-2.30) | 0.036 |  | 1.56 (1.02-2.39) | 0.041 |  | 1.00 (0.62-1.62) | 0.997 |  | 0.99 (0.59-1.68) | 0.983 |  | 1.77 (1.02-3.05) | 0.040 |  | 1.79 (1.01-3.16) | 0.047 |

Abbreviations: hazard ratio, HR; confidence interval, CI; allogeneic hematopoietic stem cell, allo-HSCT; performance status, PS; hematopoietic cell transplantation-specific comorbidity index, HCT-CI; complete remission, CR; human T-cell lymphotropic virus type I, HTLV-1; myeloablative conditioning, MAC; reduced intensity conditioning, RIC; graft-versus-host disease, GVHD.

**Supplementary Table 4. Multivariate analysis of the URD group**

|  | **Overall mortality** | | | | |  | **Relapse rate** | | | | |  | **Non-relapse mortality** | | | | |
| --- | --- | --- | --- | --- | --- | --- | --- | --- | --- | --- | --- | --- | --- | --- | --- | --- | --- |
|  | **Univariate analysis** | |  | **Multivariate analysis** | |  | **Univariate analysis** | |  | **Multivariate analysis** | |  | **Univariate analysis** | |  | **Multivariate analysis** | |
| **Variable** | **HR  (95% CI)** | ***P*** |  | **HR  (95% CI)** | ***P*** |  | **HR  (95% CI)** | ***P*** |  | **HR  (95% CI)** | ***P*** |  | **HR  (95% CI)** | ***P*** |  | **HR  (95% CI)** | ***P*** |
| **Age at allo-HSCT** |  |  |  |  |  |  |  |  |  |  |  |  |  |  |  |  |  |
| <50 years | 1.00 |  |  | - | |  | 1.00 |  |  | 1.00 |  |  | 1.00 |  |  | - | |
| ≥50 years | 1.24 (0.89-1.72) | 0.20 |  | - | |  | 1.56 (0.93-2.60) | 0.089 |  | 1.55 (0.92-2.63) | 0.100 |  | 0.95 (0.63-1.42) | 0.788 |  | - | |
| **Patient sex** |  |  |  |  |  |  |  |  |  |  |  |  |  |  |  |  |  |
| Female | 1.00 |  |  | - | |  | 1.00 |  |  | - | |  | 1.00 |  |  | *Not selected* | |
| Male | 1.22 (0.95-1.57) | 0.127 |  | - | |  | 1.10 (0.77-1.59) | 0.594 |  | - | |  | 1.33 (0.95-1.86) | 0.094 |  |  |  |
| **PS at allo-HSCT** |  |  |  |  |  |  |  |  |  |  |  |  |  |  |  |  |  |
| 0-1 | 1.00 |  |  | - | |  | 1.00 |  |  | - | |  | 1.00 |  |  | - | |
| 2-4 | 1.27 (0.73-2.23) | 0.399 |  | - | |  | 0.87 (0.35-2.16) | 0.765 |  | - | |  | 1.25 (0.61-2.58) | 0.537 |  | - | |
| **HCT-CI** |  |  |  |  |  |  |  |  |  |  |  |  |  |  |  |  |  |
| 0 | 1.00 |  |  | *Not selected* | |  | 1.00 |  |  | - | |  | 1.00 |  |  | - | |
| 1-2 | 1.23 (0.87-1.74) | 0.238 |  |  |  |  | 1.11 (0.67-1.84) | 0.693 |  | - | |  | 1.07 (0.67-1.71) | 0.787 |  | - | |
| ≥3 | 1.42 (0.98-2.06) | 0.065 |  |  |  |  | 1.39 (0.83-2.33) | 0.208 |  | - | |  | 1.13 (0.69-1.86) | 0.635 |  | - | |
| Missing | 0.78 (0.49-1.23) | 0.280 |  |  |  |  | 0.75 (0.40-1.41) | 0.370 |  | - | |  | 0.93 (0.52-1.66) | 0.805 |  | - | |
| **Disease status** |  |  |  |  |  |  |  |  |  |  |  |  |  |  |  |  |  |
| CR | 1.00 |  |  | - | |  | 1.00 |  |  | - | |  | 1.00 |  |  | - | |
| Others | 1.23 (0.94-1.60) | 0.129 |  | - | |  | 1.19 (0.82-1.72) | 0.371 |  | - | |  | 1.06 (0.75-1.51) | 0.738 |  | - | |
| **Clinical subtype** |  |  |  |  |  |  |  |  |  |  |  |  |  |  |  |  |  |
| Acute | 1.00 |  |  | - | |  | 1.00 |  |  | - | |  | 1.00 |  |  | - | |
| Others | 1.03 (0.79-1.34) | 0.821 |  | - | |  | 1.5 (0.80-1.67) | 0.444 |  | - | |  | 1.02 (0.73-1.43) | 0.910 |  | - | |
| **Sex combination** |  |  |  |  |  |  |  |  |  |  |  |  |  |  |  |  |  |
| Others | 1.00 |  |  | - | |  | 1.00 |  |  | - | |  | 1.00 |  |  | - | |
| Female donor to male recipient | 1.05 (0.73-1.52) | 0.773 |  | - | |  | 0.78 (0.43-1.40) | 0.409 |  | - | |  | 1.26 (0.81-1.9&) | 0.299 |  | - | |
| **Donor’s CMV serostatus** |  |  |  |  |  |  |  |  |  |  |  |  |  |  |  |  |  |
| Positive | 1.00 |  |  | - | |  | 1.00 |  |  | - | |  | 1.00 |  |  | - | |
| Negative | 0.99 (0.75-1.30) | 0.939 |  | - | |  | 0.80 (0.55-1.19) | 0.273 |  | - | |  | 1.15 (0.81-1.64) | 0.440 |  | - | |
| **Conditioning regimen intensity** |  |  |  |  |  |  |  |  |  |  |  |  |  |  |  |  |  |
| RIC | 1.00 |  |  | - | |  | 1.00 |  |  | - | |  | 1.00 |  |  | 1.00 |  |
| MAC | 1.23 (0.95-1.60) | 0.110 |  | - | |  | 0.91 (0.63-1.33) | 0.640 |  | - | |  | 1.46 (1.05-2.04) | 0.025 |  | 1.42 (1.01-1.98) | 0.042 |
| **GVHD prophylaxis** |  |  |  |  |  |  |  |  |  |  |  |  |  |  |  |  |  |
| CsA-based | 1.00 |  |  | - | |  | 1.00 |  |  | - | |  | 1.00 |  |  | - | |
| Tac-based | 1.04 (0.71-1.54) | 0.832 |  | - | |  | 1.76 (0.90-3.45) | 0.100 |  | - | |  | 0.79 (0.49-1.25) | 0.307 |  | - | |
| **Interval from diagnosis to allo-HSCT** |  |  |  |  |  |  |  |  |  |  |  |  |  |  |  |  |  |
| <120 days | 1.00 |  |  | - | |  | 1.00 |  |  | - | |  | 1.00 |  |  | - | |
| ≥120 days | 1.12 (0.53-2.38) | 0.763 |  | - | |  | 2.15 (0.52-8.82) | 0.290 |  | - | |  | 0.87 (0.35-2.17) | 0.768 |  | - | |
| **Years of allo-HSCT** |  |  |  |  |  |  |  |  |  |  |  |  |  |  |  |  |  |
| 2001-2011 | 1.00 |  |  | 1.00 |  |  | 1.00 |  |  | - | |  | 1.00 |  |  | 1.00 |  |
| 2012-2022 | 1.29 (0.99-1.68) | 0.055 |  | 1.30 (1.00-1.69) | 0.053 |  | 0.92 (0.64-1.33) | 0.667 |  | - | |  | 1.34 (0.95-1.90) | 0.094 |  | 1.35 (0.96-1.92) | 0.087 |
| **Acute GVHD** |  |  |  |  |  |  |  |  |  |  |  |  |  |  |  |  |  |
| No | 1.00 |  |  | 1.00 |  |  | 1.00 |  |  | 1.00 |  |  | 1.00 |  |  | 1.00 |  |
| Grade I | 0.93 (0.64-1.34) | 0.684 |  | 0.87 (0.60-1.26) | 0.462 |  | 1.01 (0.61-1.65) | 0.976 |  | 1.09 (0.66-1.80) | 0.734 |  | 0.81 (0.48-1.35) | 0.416 |  | 0.75 (0.44-1.26) | 0.278 |
| Grade II-IV | 1.34 (0.98-1.82) | 0.065 |  | 1.24 (0.91-1.70) | 0.176 |  | 0.98 (0.63-1.52) | 0.934 |  | 1.03 (0.65-1.63) | 0.907 |  | 1.59 (1.06-2.40) | 0.026 |  | 1.41 (0.94-2.13) | 0.099 |
| **Chronic GVHD** |  |  |  |  |  |  |  |  |  |  |  |  |  |  |  |  |  |
| No | 1.00 |  |  | - | |  | 1.00 |  |  | 1.00 |  |  | 1.00 |  |  | *Not selected* | |
| Limited type | 0.70 (0.44-1.09) | 0.115 |  | - | |  | 0.65 (0.33-1.31) | 0.229 |  | 0.66 (0.33-1.31) | 0.234 |  | 1.25 (0.74-2.10) | 0.407 |  |  |  |
| Extensive type | 1.01 (0.75-1.36) | 0.956 |  | - | |  | 0.56 (0.33-0.93) | 0.027 |  | 0.57 (0.34-0.95) | 0.030 |  | 2.13 (1.50-3.02) | <0.001 |  |  |  |
| **CMV reactivation** |  |  |  |  |  |  |  |  |  |  |  |  |  |  |  |  |  |
| No | 1.00 |  |  | 1.00 |  |  | 1.00 |  |  | 1.00 |  |  | 1.00 |  |  | 1.00 |  |
| Yes | 1.52 (1.05-2.18) | 0.025 |  | 1.45 (1.00-2.09) | 0.048 |  | 1.08 (0.67-1.74) | 0.746 |  | 1.16 (0.71-1.91) | 0.552 |  | 1.84 (1.10-3.07) | 0.020 |  | 1.68 (1.01-2.82) | 0.048 |

**Supplementary Table 5. Multivariate analysis of the U-CB group**

|  | **Overall mortality** | | | | |  | **Relapse rate** | | | | |  | **Non-relapse mortality** | | | | |
| --- | --- | --- | --- | --- | --- | --- | --- | --- | --- | --- | --- | --- | --- | --- | --- | --- | --- |
|  | **Univariate analysis** | |  | **Multivariate analysis** | |  | **Univariate analysis** | |  | **Multivariate analysis** | |  | **Univariate analysis** | |  | **Multivariate analysis** | |
| **Variable** | **HR  (95% CI)** | ***P*** |  | **HR  (95% CI)** | ***P*** |  | **HR  (95% CI)** | ***P*** |  | **HR  (95% CI)** | ***P*** |  | **HR  (95% CI)** | ***P*** |  | **HR  (95% CI)** | ***P*** |
| **Age at allo-HSCT** |  |  |  |  |  |  |  |  |  |  |  |  |  |  |  |  |  |
| <50 years | 1.00 |  |  | - | |  | 1.00 |  |  | - | |  | 1.00 |  |  | 1.00 |  |
| ≥50 years | 1.27 (0.83-1.95) | 0.264 |  | - | |  | 0.76 (0.47-1.24) | 0.266 |  | - | |  | 2.38 (1.10-5.15) | 0.027 |  | 2.80 (1.26-6.20) | 0.011 |
| **Patient sex** |  |  |  |  |  |  |  |  |  |  |  |  |  |  |  |  |  |
| Female | 1.00 |  |  | 1.00 |  |  | 1.00 |  |  | - | |  | 1.00 |  |  | - | |
| Male | 1.32 (0.97-1.79) | 0.077 |  | 1.33 (0.97-1.82) | 0.079 |  | 1.26 (0.85-1.87) | 0.260 |  | - | |  | 1.24 (0.80-1.92) | 0.341 |  | - | |
| **PS at allo-HSCT** |  |  |  |  |  |  |  |  |  |  |  |  |  |  |  |  |  |
| 0-1 | 1.00 |  |  | - | |  | 1.00 |  |  | - | |  | 1.00 |  |  | - | |
| 2-4 | 1.55 (0.79-3.04) | 0.203 |  | - | |  | 0.82 (0.31-2.13) | 0.680 |  | - | |  | 1.58 (0.65-3.82) | 0.310 |  | - | |
| **HCT-CI** |  |  |  |  |  |  |  |  |  |  |  |  |  |  |  |  |  |
| 0 | 1.00 |  |  | - | |  | 1.00 |  |  | - | |  | 1.00 |  |  | 1.00 |  |
| 1-2 | 1.31 (0.88-1.93) | 0.179 |  | - | |  | 1.02 (0.62-1.67) | 0.933 |  | - | |  | 1.43 (0.82-2.52) | 0.208 |  | 1.38 (0.79-2.41) | 0.258 |
| ≥3 | 1.25 (0.71-2.19) | 0.435 |  | - | |  | 0.42 (0.14-1.22) | 0.111 |  | - | |  | 2.37 (1.22-4.60) | 0.011 |  | 2.14 (0.95-4.83) | 0.067 |
| Missing | 1.11 (0.67-1.84) | 0.674 |  | - | |  | 0.81 (0.41-1.57) | 0.529 |  | - | |  | 1.34 (0.66-2.70) | 0.415 |  | 1.58 (0.75-3.33) | 0.232 |
| **Disease status** |  |  |  |  |  |  |  |  |  |  |  |  |  |  |  |  |  |
| CR | 1.00 |  |  | - | |  | 1.00 |  |  | - | |  | 1.00 |  |  | - | |
| Others | 1.02 (0.74-1.41) | 0.887 |  | - | |  | 1.29 (0.86-1.93) | 0.217 |  | - | |  | 0.74 (0.46-1.18) | 0.206 |  | - | |
| **Clinical subtype** |  |  |  |  |  |  |  |  |  |  |  |  |  |  |  |  |  |
| Acute | 1.00 |  |  | - | |  | 1.00 |  |  | - | |  | 1.00 |  |  | - | |
| Others | 0.93 (0.67-1.2)) | 0.662 |  | - | |  | 0.93 (0.61-1.42) | 0.749 |  | - | |  | 0.93 (0.58-1.48) | 0.752 |  | - | |
| **Sex combination** |  |  |  |  |  |  |  |  |  |  |  |  |  |  |  |  |  |
| Others | 1.00 |  |  | - | |  | 1.00 |  |  | 1.00 |  |  | 1.00 |  |  | - | |
| Female donor to male recipient | 1.15 (0.81-1.62) | 0.434 |  | - | |  | 1.46 (0.96-2.23) | 0.077 |  | 1.49 (0.97-2.30) | 0.070 |  | 0.86 (0.52-1.42) | 0.555 |  | - | |
| **Conditioning regimen intensity** |  |  |  |  |  |  |  |  |  |  |  |  |  |  |  |  |  |
| RIC | 1.00 |  |  | - | |  | 1.00 |  |  | - | |  | 1.00 |  |  | 1.00 |  |
| MAC | 1.17 (0.85-1.60) | 0.342 |  | - | |  | 0.73 (0.48-1.13) | 0.158 |  | - | |  | 1.54 (0.99-2.40) | 0.055 |  | 1.67 (1.03-2.71) | 0.037 |
| **GVHD prophylaxis** |  |  |  |  |  |  |  |  |  |  |  |  |  |  |  |  |  |
| CsA-based | 1.00 |  |  | 1.00 |  |  | 1.00 |  |  | - | |  | 1.00 |  |  | - | |
| Tac-based | 1.04 (0.74-1.44) | 0.836 |  | 1.00 (0.71-1.40) | 0.987 |  | 1.32 (0.85-2.04) | 0.215 |  | - | |  | 0.92 (0.57-1.46) | 0.718 |  | - | |
| **Interval from diagnosis to allo-HSCT** |  |  |  |  |  |  |  |  |  |  |  |  |  |  |  |  |  |
| <120 days | 1.00 |  |  | - | |  | 1.00 |  |  | - | |  | 1.00 |  |  | - | |
| ≥120 days | 0.98 (0.71-1.36) | 0.910 |  | - | |  | 0.92 (0.61-1.40) | 0.708 |  | - | |  | 1.24 (0.77-1.99) | 0.379 |  | - | |
| **Years of allo-HSCT** |  |  |  |  |  |  |  |  |  |  |  |  |  |  |  |  |  |
| 2001-2011 | 1.00 |  |  | - | |  | 1.00 |  |  | - | |  | 1.00 |  |  | - | |
| 2012-2022 | 0.96 (0.69-1.32) | 0.784 |  | - | |  | 1.03 (0.68-1.57) | 0.870 |  | - | |  | 1.08 (0.69-1.70) | 0.735 |  | - | |
| **Acute GVHD** |  |  |  |  |  |  |  |  |  |  |  |  |  |  |  |  |  |
| No | 1.00 |  |  | 1.00 |  |  | 1.00 |  |  | 1.00 |  |  | 1.00 |  |  | 1.00 |  |
| Grade I | 0.72 (0.47-1.09) | 0.117 |  | 0.70 (0.45-1.07) | 0.102 |  | 0.96 (0.59-1.56) | 0.869 |  | 0.84 (0.51-1.40) | 0.507 |  | 0.60 (0.31-1.17) | 0.131 |  | 0.52 (0.25-1.07) | 0.075 |
| Grade II-IV | 1.11 (0.79-1.58) | 0.539 |  | 1.09 (0.75-1.60) | 0.647 |  | 0.54 (0.34-0.87) | 0.011 |  | 0.50 (0.30-0.81) | 0.005 |  | 1.62 (0.99-2.64) | 0.055 |  | 1.41 (0.83-2.39) | 0.206 |
| **Chronic GVHD** |  |  |  |  |  |  |  |  |  |  |  |  |  |  |  |  |  |
| No | 1.00 |  |  | 1.00 |  |  | 1.00 |  |  | - | |  | 1.00 |  |  | *Not selected* | |
| Limited type | 0.50 (0.28-0.91) | 0.023 |  | 0.51 (0.28-0.93) | 0.028 |  | 0.86 (0.44-1.69) | 0.668 |  | - | |  | 0.63 (0.27-1.46) | 0.279 |  |  |  |
| Extensive type | 1.07 (0.69-1.66) | 0.762 |  | 0.97 (0.61-1.54) | 0.881 |  | 0.63 (0.31-1.26) | 0.190 |  | - | |  | 1.89 (1.09-3.27) | 0.024 |  |  |  |
| **CMV reactivation** |  |  |  |  |  |  |  |  |  |  |  |  |  |  |  |  |  |
| No | 1.00 |  |  | 1.00 |  |  | 1.00 |  |  | 1.00 |  |  | 1.00 |  |  | 1.00 |  |
| Yes | 1.42 (0.94-2.13) | 0.092 |  | 1.34 (0.88-2.03) | 0.177 |  | 1.43 (0.85-2.39) | 0.179 |  | 1.56 (0.92-2.66) | 0.100 |  | 1.36 (0.75-2.44) | 0.310 |  | 1.16 (0.62-2.19) | 0.646 |
